# Management of Post-traumatic Ear Reconstruction in a Tertiary Care Center: A Retrospective Study

**DOI:** 10.7759/cureus.105893

**Published:** 2026-03-26

**Authors:** Vijaykumar Huded, Gazal Gautam

**Affiliations:** 1 General Surgery, BLDE (Deemed to Be University), Shri B. M. Patil Medical College, Hospital and Research Centre, Vijayapura, IND; 2 Plastic and Reconstructive Surgery, Institute of Medical Sciences, Banaras Hindu University, Varanasi, IND; 3 Hand and Microvascular Surgery, Ganga Hospital, Coimbatore, IND

**Keywords:** acquired auricular deformity, auricular trauma, costal cartilage graft, ear avulsion, postauricular flap, post-traumatic auricular reconstruction, road traffic accidents

## Abstract

Background

The external ear, due to its prominent anatomical position and complex three-dimensional (3D) cartilaginous framework, is particularly susceptible to traumatic injuries. There has been a rising incidence of partial and total auricular deformities resulting from road traffic accidents, assaults, human and animal bites, burns, and accidental falls. These injuries are often aesthetically conspicuous and psychologically distressing. Reconstruction of post-traumatic auricular defects poses significant challenges because of the need to restore bilateral symmetry, the limited availability of adjacent skin, and the presence of the external auditory canal. Although various reconstructive techniques have been described, including the Baudet method, pocket principle, microvascular reattachment, local flaps, composite grafting, and emerging modalities such as 3D printing, no standardized treatment guidelines currently exist.

Methodology

This retrospective study analyzed 25 patients treated for acquired auricular deformities at a tertiary care center for a period of two years, from 2023 to 2025, and included all patients with acquired ear deformity and injuries. Data were collected regarding patient demographics, mechanism of injury, anatomical location and zone of defect, reconstructive technique employed, number of surgical stages, and postoperative complications. The study aimed to identify the most suitable reconstructive approach based on defect characteristics and to evaluate the advantages and limitations of different techniques.

Results

Road traffic accidents were the most common cause of injury (56%), followed by assault (24%), bite injuries (8%), self-falls (4%), and burns (4%). The upper third of the auricle was the most frequently affected region (36%), followed by the middle third (12%), combined upper and middle thirds (12%), lower third (4%), and total ear involvement (4%). Costal cartilage grafting was required in 8% of cases. Postauricular skin flaps were the most commonly utilized method for soft tissue coverage (20%). The findings align with existing literature, confirming road traffic accidents as the predominant cause of auricular trauma and the upper third as the most commonly involved region. The protocol suggested in our study is easy to practice and can be performed by newly graduated plastic surgeons, resulting in better results.

Conclusions

Post-traumatic auricular reconstruction requires individualized planning based on defect location, extent of tissue loss, and condition of surrounding skin. Despite the availability of multiple reconstructive techniques, no single method is universally applicable. A tailored approach yields satisfactory functional and aesthetic outcomes. Further studies are required to establish standardized management guidelines for auricular avulsion injuries.

## Introduction

The external ear, owing to its peculiar position on the side of the head and its protruding anatomical structure, is particularly vulnerable to traumatic injuries such as road traffic accidents, physical assaults, human and animal bites, burns, and accidental falls [1,2]. The ear’s complex three-dimensional (3D) cartilage framework and elastic nature make such injuries particularly conspicuous and often psychologically distressing to affected individuals, regardless of their severity. Reconstruction poses a unique challenge due to the bilateral symmetry of the ear and limited availability of adjacent skin, especially in the presence of a pre-existing external auditory canal [3].

Effective post-traumatic auricular reconstruction requires careful evaluation concerning anatomical location, zone of the defect, missing components, and condition of the surrounding skin to determine the most appropriate surgical approach [4]. Despite the availability of multiple surgical techniques for managing auricular avulsion, such as the Baudet method, the pocket principle, microvascular reattachment, reconstruction with local flaps, composite grafting, and emerging technologies such as 3D printing, there are currently no standardized treatment guidelines [5]. This lack of consensus presents challenges in selecting the optimal approach for individual cases. The standardization has been difficult because of the nature of injuries involving various parts of the ear and surrounding skin. Depending upon the part of the ear involved as well as the condition of the surrounding skin, reconstructive techniques are decided upon.

This study presents a retrospective analysis of 25 cases treated at a tertiary care center for acquired auricular deformities to provide an overview of the type of injuries and applied reconstructive techniques, with the primary objective being reconstruction of the injured ear and trying to achieve a near-normal appearing ear, and the secondary objective being to help patients integrate back into society with self-confidence. Data were collected regarding patient demographics, mechanism of injury, anatomical site of damage, zone of injury, and the technique opted for, along with the number of stages of reconstruction and the complications. This study mainly aims to compare various techniques and their outcomes to benefit the patient.

Road traffic accidents accounted for the majority of cases, followed by assault, bite injuries, self-falls, and burns. The upper third of the auricle was most frequently involved, followed by injuries to the middle third, both upper and middle third, lower third, and, least commonly, to the entire ear. Post-auricular skin flaps were the most commonly employed technique of reconstruction. The findings are consistent with existing literature, reaffirming road traffic accidents as the leading cause of acquired auricular trauma and the upper third of the ear as the most commonly affected region.

## Materials and methods

This retrospective study was conducted at BLDE (Deemed to Be University) Shri B. M. Patil Medical College, Hospital and Research Centre, Vijayapura, after obtaining approval from the Institutional Ethics Committee (approval number: BLDE (DU)/IEC/1177//2025-26). Data were collected from January 2023 to January 2025 from medical records as well as follow-up of patients. All consecutive patients with ear injuries or acquired ear deformities during the study period were included after obtaining written informed consent. Acquired ear deformities were defined as any structural abnormalities of the external ear that developed after birth due to external injuries such as road traffic accidents, assault, bite, burns, and piercing. Patients with congenital ear deformities or tumorous lesions of the ear were excluded from the study.

Each injury was classified according to anatomical region, designated as the upper third, middle third, lower third, or a combination thereof, allowing for direct comparison with previously published data. This simple classification was done to simplify our management protocol; hence, it was not compared to a currently existing classification system. The following characteristics were recorded: name, gender, age, laterality, mode of injury, defect, anatomical region, zones of injury, technique employed, number of stages of reconstruction, associated complications, and the overall outcome, as presented in Table 1.

**Table 1:** Demographic details, mode and site of injury, management, and outcome of treatment. M = male; F = female; R = right; L = left; B/L = bilateral; RTA = road traffic accident; C/L = contralateral; FTSG = full-thickness skin grafting

Serial number	Age	Sex	Laterality	Mode of injury	Diagnosis	Defect	Anatomical region	Technique of management	Number of stages	Outcome	Complications
1	23	M	R	RTA	Right ear near-total amputation	Composite	Upper third	Ear reconstruction	1	Excellent	Nil
2	32	M	R	RTA	Right ear near-total amputation (4 days old)	Composite	Middle third	Debridement with cartilage repair	1	Good	Perichondritis
3	26	M	R	Assault with a sharp object	Laceration over the right ear	Composite	Upper and middle	Cartilage reconstruction with primary repair	1	Excellent	Nil
4	40	M	R	RTA	Scalp avulsion with lip laceration and ear laceration	Skin	Lower third	Primary repair	1	Excellent	Nil
5	17	M	L	Assault with a sharp object	Left ear near-total amputation with scalp laceration	Composite	Upper and middle	Reconstruction with primary repair	1	Excellent	Nil
6	34	M	B/L	RTA	B/L ear crush injury	Composite	Upper and middle	Superior-based post-auricular flap, inferior-based post-auricular flap with contralateral ear cartilage graft	1	Good	Cauliflower ear deformity
7	35	M	R	RTA	Right ear crush injury	Composite	Lower third	Debridement with post-auricular coverage	2	Excellent	Nil
8	27	M	R	RTA	Right ear avulsion with nasal area and philtrum laceration with forehead laceration	Composite	Upper third	Debridement with primary repair	1	Excellent	Nil
9	23	M	L	Assault with a sharp object	Near-total left ear avulsion	Composite	Middle third	Debridement with primary repair of cartilage and skin	1	Excellent	Nil
10	25	M	L	RTA	Upper helix and antihelix loss of the right ear	Composite	Upper and middle	Ear reconstruction with post-auricular flap	2	Excellent	Nil
11	32	M	R	RTA	Laceration over the right ear and the right supraorbital region	Skin	Upper third	Debridement with primary suturing	1	Excellent	Nil
12	17	F	R	RTA	Right ear near-total amputation	Composite	Upper and middle	Debridement with primary suturing	1	Excellent	Nil
13	6	M	R	RTA	Right ear helical laceration	Skin	Middle third	Debridement with primary repair	1	Excellent	Nil
14	60	M	L	Assault with a sharp object	Post-traumatic ear transection with cheek laceration	Composite	Upper third	Debridement with ear reconstruction	1	Excellent	Nil
15	45	M	L	RTA	Right foot crush injury with ear laceration	Skin	Middle third	Debridement with primary repair	1	Excellent	Nil
16	50	M	R	Assault with a sharp object	Scalp avulsion with right ear laceration	Composite	Upper third	Debridement with primary repair	1	Excellent	Nil
17	29	M	R	Human bite	Right ear helical rim defect	Composite	Upper third	Debridement with helical rim reconstruction with post-auricular flap	2	Excellent	Distal tip flap necrosis
18	30	M	R	RTA	Right ear avulsion injury	Composite	Upper third	Debridement with ear reconstruction	1	Excellent	Nil
19	56	M	R	Fall over a wooden piece	Complete avulsion of the upper one-third of the ear	Composite	Upper third	Primary repair	1	Excellent	Nil
20	35	F	R	RTA	Post-traumatic ear transection with cheek laceration	Composite	All 3 zones	C/L chondrocutaneous graft with FTSG coverage	1	Satisfactory	Nil
21	26	M	R	RTA	Post-traumatic upper helix deformity	Composite	Upper third	Autologous rib cartilage graft	1	Excellent	Nil
22	28	M	R	Human bite	Loss of helix	Composite	Middle and lower	Post-auricular flap reconstruction	2	Excellent	Nil
23	56	M	R	Assault with a sharp object	Upper one-third helix loss	Composite	Upper third	Primary repair	1	Excellent	Nil
24	30	F	R	Post-ear piercing	Keloid formation	Skin	Upper third	Excision and coverage with a post-auricular flap	2	Excellent	Nil
25	22	F	R	Post-burn	Right ear deformity	Composite	Upper and middle	Costochondral rib cartilage graft with temporalis fascia coverage with FTSG	1	Excellent	Nil

The patients were followed up postoperatively every four days for the first 15 days, then every week for the next 15 days, followed by monthly once for the next one and a half months, making a total of three months of follow-up period. The staged procedures had a gap of 21 days or three weeks between them.

If only tissue loss was present with intact cartilage, a post-auricular flap was used. If both tissue and cartilage were lost, a cartilage graft with flap reconstruction was opted for. If the entire ear was lost, costochondral reconstruction was performed, and for lacerations, primary repair was done. Patient-reported outcomes were measured using a standardized questionnaire with overall satisfaction rated on a five-point Likert scale at the end of the three-month follow-up period, as shown in Table 2. Anonymous feedback was also permitted.

**Table 2:** Five-point Likert scale describing patient satisfaction for the overall outcome.

Score	Satisfaction level
1	Very dissatisfied
2	Dissatisfied
3	Satisfactory
4	Good
5	Excellent

## Results

**Figure 1:**
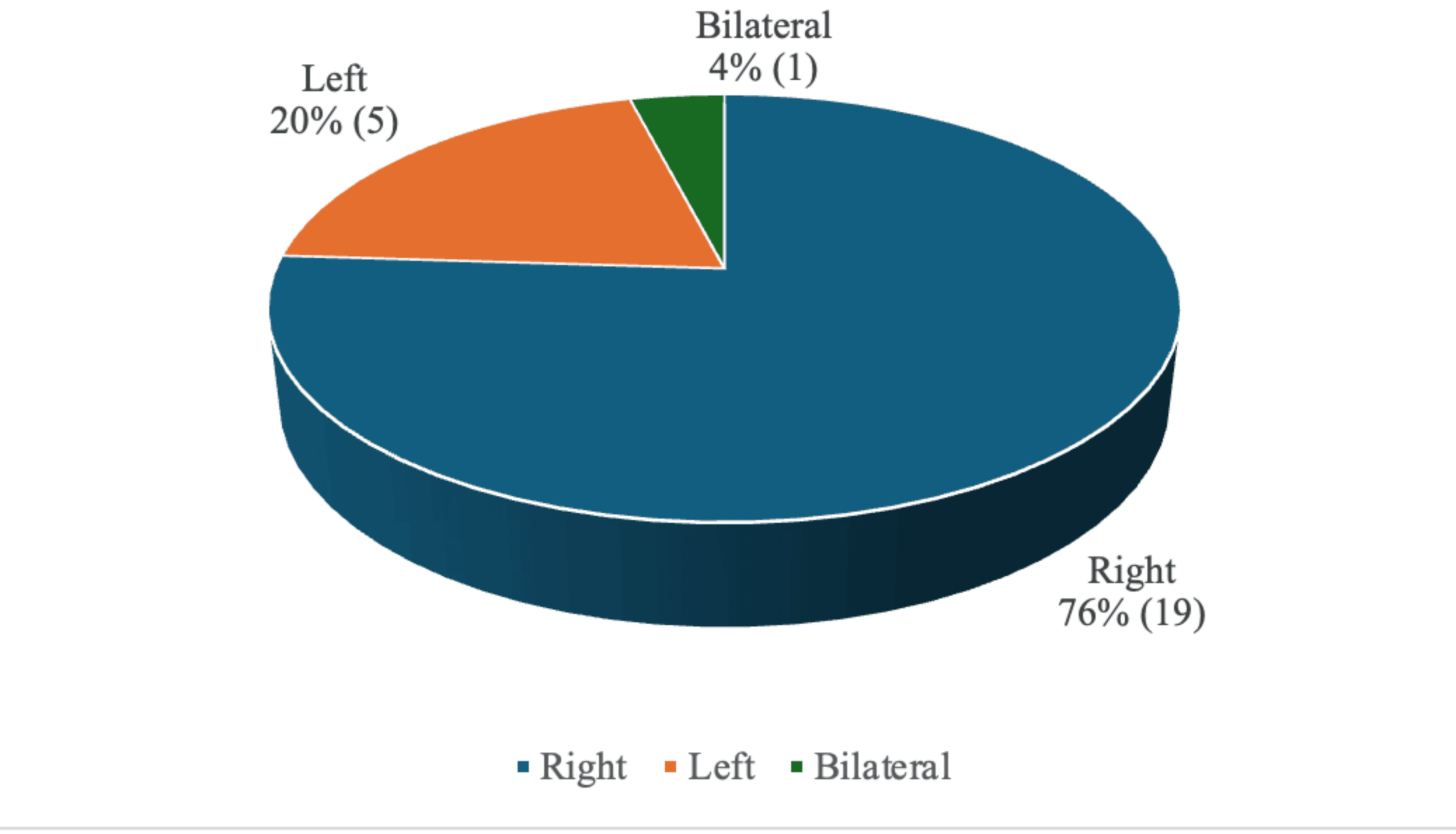
Laterality of the ear affected.

A total of 25 patients, four (16%) females and 21 (84%) males, were included, and their records were analyzed, with a male-to-female ratio of 5.25:1. The observed male predominance may be attributed to increased involvement of males in outdoor activities, occupational exposure, and a higher incidence of trauma-related injuries. The age of the patients ranged from 6 to 60 years, with a mean age in the female group of 26 years and 33.33 years in the male group, indicating a slightly higher age distribution in the male cohort. The right ear was affected in 19 (76%) cases, five (20%) patients had a left ear injury, and one (4%) patient had a bilateral ear injury, as can be seen in Figure 1.

**Figure 2:**
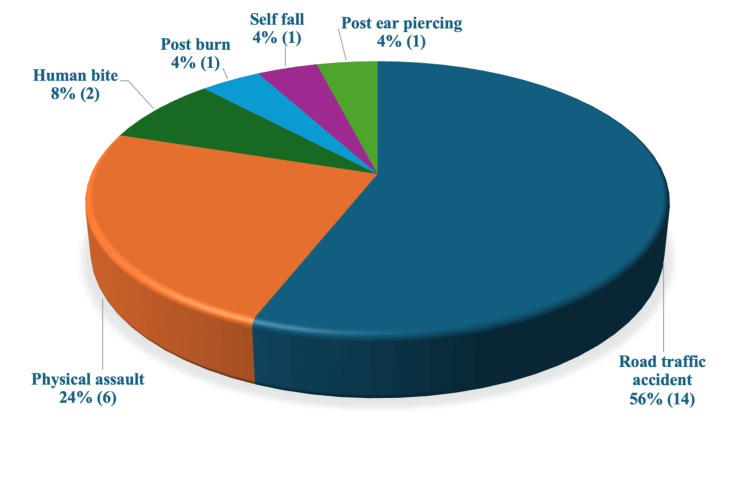
Distribution of the mode of injury.

The injuries were caused by road traffic accidents (14), physical assaults (6), post-human bite (2), post-burns (1), self-fall (1), and post-ear piercing (1). Only six (24%) patients had an injury involving a single isolated zone, whereas 19 (76%) patients had a combination of two or more zones injured, as shown in Figure 2.

**Figure 3:**
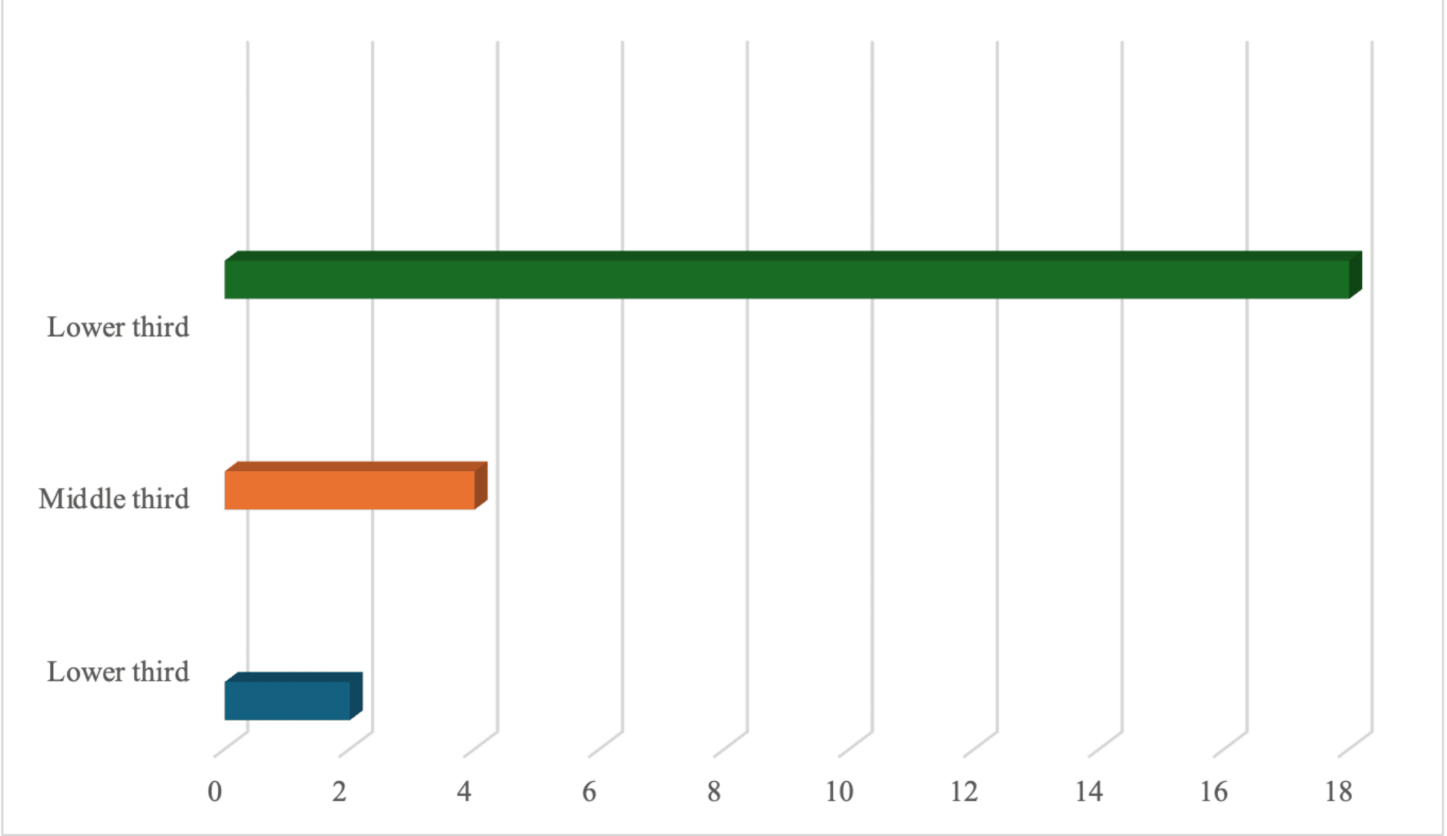
Distribution of patients according to the zone of ear affected.

The upper third of the auricle was the most often affected by traumatic injury (n = 9; 36%). In another six cases, the uppermost two-thirds of the auricle was affected. One ear was lost completely and required total auricular reconstruction. Injuries of the middle third (n = 4), lower third (n = 2), and lower two-thirds (n = 1) were seen less frequently, as shown in Figure 3.

Primary repair was done in 10 patients, a post-auricular flap was used for reconstruction in five patients, a temporalis fascia flap with full-thickness skin grafting (FTSG) was used in one patient, and a contralateral chondrocutaneous graft with FTSG coverage was used in one patient. Autologous rib cartilage graft was utilized only in patients < 30 years old, as in the older age group, the costochondral junction becomes hard, making it difficult to harvest and carve the ear framework.

**Figure 4:**
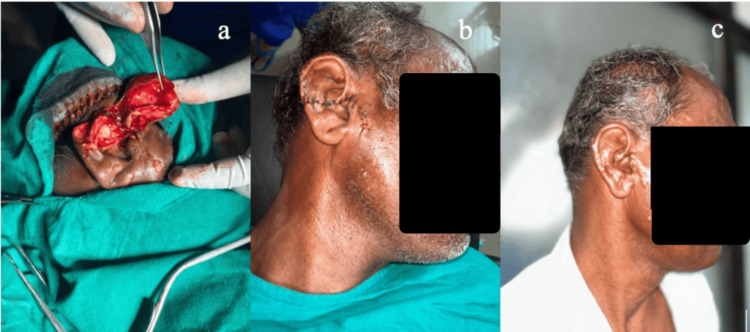
(a) Patient number 19 as noted in Table 1: A 56-year-old male who had a fall over wooden piece leading to an avulsion injury of the upper one-third of the right ear. (b) Primary repair completed. (c) Postoperative day 15.

**Figure 5:**
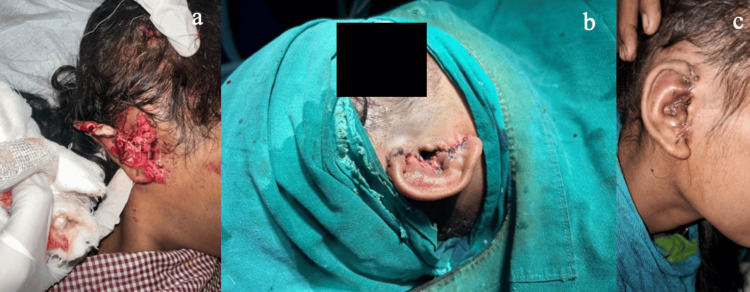
(a) Patient number 12 from Table 1: A 17-year-old female who suffered from a road traffic accident leading to the near-total amputaion of the right ear involving the upper and middle lobes. (b) Primary repair completed. (c) After suture removal.

**Figure 6:**
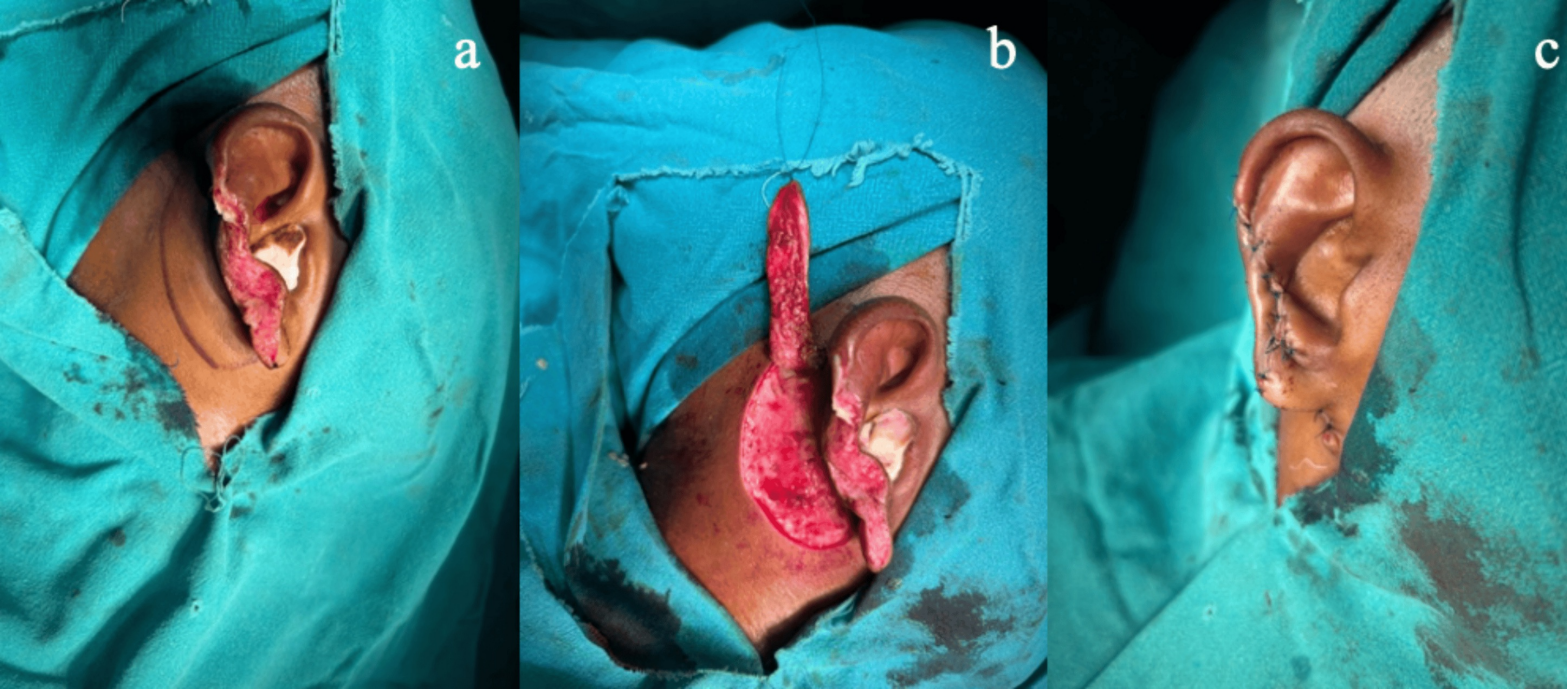
(a) Patient number 22 from Table 1: A 28-year-old male with a human bite over the right ear with the loss of the middle and lower lobes of the ear. (b) Post-auricular flap raised. (c) Flap insetting.

**Figure 7:**
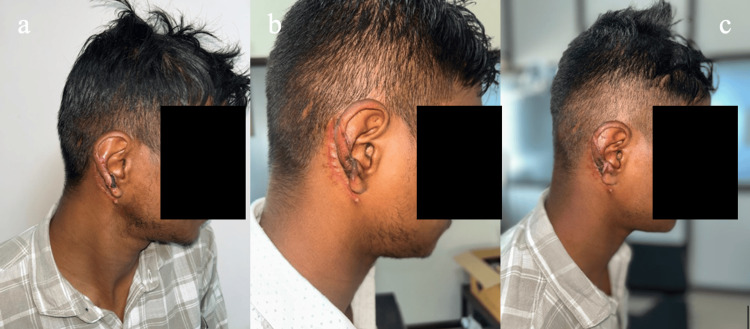
Same patient as in Figure 6: (a) Mild distal flap necrosis. (b, c) Nicely healed flap after division.

**Figure 8:**
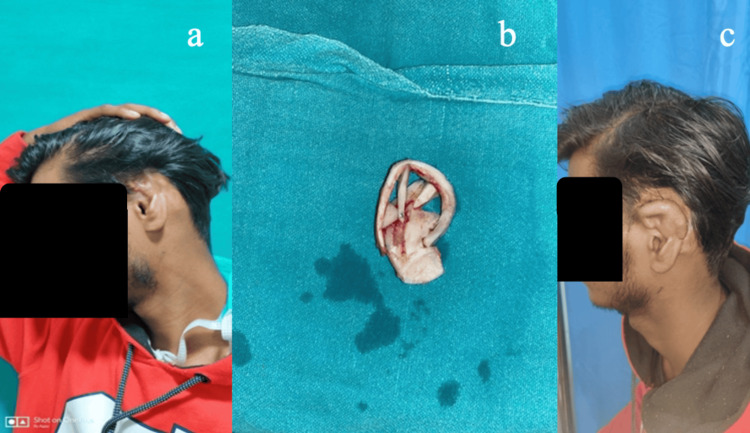
(a) Patient number 10 from Table 1: A 25-year-old male who suffered a road traffic accident involving the loss of the upper helix and antihelix of the left ear. (b) Ear framework made of costochondral junction. (c) Final appearance after elevation of framework.

**Figure 9:**
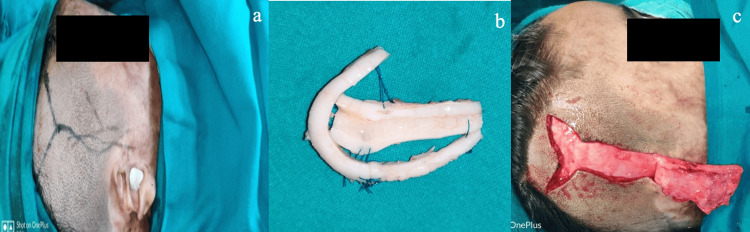
(a) Patient number 25 from Table 1: A 22-year-old female who presented with a post-burn loss of the upper and middle one-third of the right ear. (b) Ear framework made of costochondral cartilage. (c) Temporalis fascial flap coverage of the ear cartilage framework.

**Figure 10:**
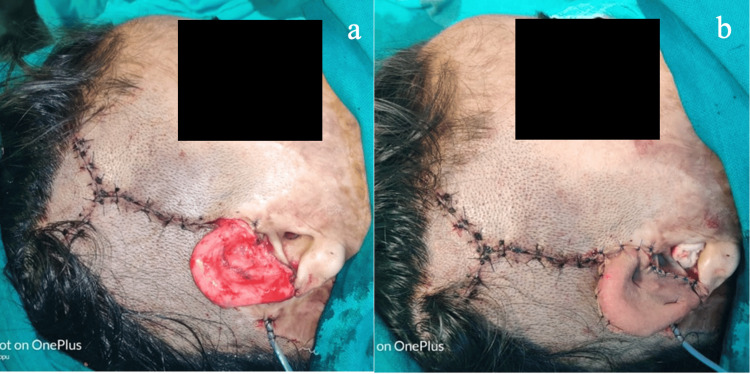
Same patient as in Figure 9. (a) Temporal fascial flap coverage over the ear framework. (b) Split-thickness skin graft coverage over the temporalis fascial flap.

The temporoparietal fascial flap covering the cartilage framework needed a single-stage reconstruction, and it was also found to be useful when the surrounding area was scarred. Three patients developed complications of perichondritis, cauliflower ear deformity, and distal flap necrosis, while the remaining 22 patients had an excellent outcome, as per the patient satisfaction Likert score, in terms of satisfactory contour, patient satisfaction, and overall aesthetic. Some of the notable examples operated in the study are presented in Figures 4-10.

## Discussion

Auricular reconstruction is regarded as one of the most challenging procedures in reconstructive surgery, primarily due to the complex architecture of the auricular cartilage and the fragile nature of its overlying soft tissue envelope. In our study, road traffic accidents emerged as the most frequent cause of post-traumatic ear deformities at 56% (n = 14). This finding aligns with previous literature: Harris et al. reported traffic-related auricular injuries in 18% (n = 5) of cases [6], Pearl and Sabbagh observed a rate of 12% (n = 6) [7], while Hyckel et al. documented a notably higher incidence of 87% (n = 13) [8].

The higher prevalence observed in our study likely reflects region-specific factors, particularly the widespread use of two-wheelers, increased exposure to high-velocity road traffic accidents, and inconsistent use of helmets, especially helmets lacking adequate lateral ear protection. Additionally, disparities in inclusion criteria across studies may account for the observed differences; for instance, studies focusing on partial auricular amputations or severe traumatic defects are more likely to report higher proportions of road traffic-related injuries. Differences in healthcare infrastructure and referral patterns may also contribute, as tertiary care centers in developing regions often receive a greater number of high-energy trauma cases. Furthermore, temporal differences between studies and evolving road safety regulations may influence the reported incidence.

Bite injuries accounted for only 8% (n = 2) of cases in our study, a significantly lower incidence compared to findings from previous research. Pearl and Sabbagh reported 72% (36 out of 50 cases) over four years [7], while Harris et al. identified bite-related injuries in 50% (14 out of 28 cases) [6]. In a study by Henry et al., bite injuries were identified as the leading cause of acquired auricular defects. Furthermore, the ear was shown to be the most commonly affected site in facial bite injuries, surpassing even the nose, another prominent facial extremity [9]. The incidence of auricular burn injuries in our study was 4%, which is notably lower than the rates reported by Harris et al. (15%) [6], Gault (12%) [1], and Pearl and Sabbagh (10%) [7]. Upper third injuries of the auricle were, by far, the most common, followed by upper two-thirds involvement.

Although our study did not utilize a formal zonal classification system for auricular injuries, the novel classification proposed by Kumar and Jain could have provided a standardized framework for anatomical assessment. This system divides the ear into four equal zones, each delineated at a 45-degree angle and measuring approximately 2-2.5 cm per zone, and has been shown to facilitate planning of auricular reconstruction and consistent documentation in both emergency and outpatient settings [10]. Had this system been applied in our series, it would have potentially allowed for a more precise localization of injuries, enabling comparison of injury distribution across zones, and may have enhanced the reproducibility and clarity of our findings. It may have also allowed for a better reconstruction protocol.

Nonetheless, the majority of injuries in our cohort were observed in the upper and middle third of the ear, with the lower third being the least affected. One patient presented with complete auricular loss. A post-auricular flap was used in 20% (n = 5) of cases. Three patients developed complications of perichondritis, cauliflower ear deformity, and distal flap necrosis, but the majority of patients, 88% (n = 22), had an excellent outcome.

## Conclusions

Auricular injuries, although not life-threatening, have significant aesthetic and psychosocial implications due to the prominent and complex structure of the external ear. Successful reconstruction relies on early assessment, preservation of viable tissue, and the selection of an appropriate technique tailored to the defect characteristics. Based on our findings, a defect-oriented, region-specific approach can simplify decision-making in clinical practice. For defects involving the upper and middle thirds of the auricle, a superiorly based post-auricular flap provides reliable coverage with good color and texture match. In cases limited to soft tissue loss, primary repair may be sufficient; however, when cartilage loss is present, simultaneous cartilage grafting is essential to maintain structural support and prevent contour deformities. For lower third defects, an inferiorly based postauricular flap offers better reach and vascular reliability, making it the preferred option. From a practical standpoint, clinicians should prioritize: (1) early debridement with maximal preservation of viable cartilage, (2) careful assessment of defect depth (skin alone vs. skin with cartilage loss), and (3) selection of flap orientation based on defect location to ensure optimal vascularity and aesthetic outcome. Our study emphasizes that adopting a simple algorithm based on defect location and tissue involvement can lead to predictable, reproducible, and satisfactory results in auricular reconstruction. Larger multicentric studies are needed to further validate and standardize these decision-making protocols.
